# The novel P330L pathogenic variant of aromatic amino acid decarboxylase maps on the catalytic flexible loop underlying its crucial role

**DOI:** 10.1007/s00018-022-04343-w

**Published:** 2022-05-20

**Authors:** Giovanni Bisello, Katarzyna Kusmierska, Marcel M. Verbeek, Jolanta Sykut–Cegielska, Michèl A. A. P. Willemsen, Ron A. Wevers, Krystyna Szymańska, Jarosław Poznanski, Jakub Drozak, Katarzyna Wertheim–Tysarowska, Agnieszka Magdalena Rygiel, Mariarita Bertoldi

**Affiliations:** 1 Department of Neuroscience, Biomedicine and Movement Sciences, Section of Biochemistry, University of Verona, Strada Le Grazie 8, 37134 Verona, Italy; 2 Department of Screening and Metabolic Diagnostics, Institute of Mother and Child, Warsaw, Poland; 3 Department of Neurology, Donders Institute for Brain, Cognition and Behaviour, Radboud University Medical Centre, Geert Grooteplein 10, 6525 GA Nijmegen, The Netherlands; 4 Translational Metabolic Laboratory, Department Laboratory Medicine, Radboud University Medical Cente, Geert Grooteplein 10, 6525 GA Nijmegen, The Netherlands; 5 Department of Inborn Errors of Metabolism and Paediatrics, Institute of Mother and Child, Warsaw, Poland; 6 Department of Pediatric Neurology, Radboud University Medical Centre, Geert Grooteplein 10, 6525 GA Nijmegen, The Netherlands; 7 Department of Child and Adolescent Psychiatry, Medical University of Warsaw, Warsaw, Poland; 8 Department of Biophysics, Institute of Biochemistry and Biophysics, Polish Academy of Sciences, Warsaw, Poland; 9 Department of Metabolic Regulation, Faculty of Biology, University of Warsaw, Warsaw, Poland; 10 Department of Medical Genetics, Institute of Mother and Child, Warsaw, Poland

**Keywords:** Pyridoxal 5’-phosphate, Aromatic amino acid decarboxylase, Aromatic amino acid decarboxylase deficiency, Monoamine neurotransmitter disorder, Catalytic loop, Structure and function studies

## Abstract

Aromatic amino acid decarboxylase (AADC) deficiency is a rare monogenic disease, often fatal in the first decade, causing severe intellectual disability, movement disorders and autonomic dysfunction. It is due to mutations in the gene coding for the AADC enzyme responsible for the synthesis of dopamine and serotonin. Using whole exome sequencing, we have identified a novel homozygous c.989C > T (p.Pro330Leu) variant of AADC causing AADC deficiency. Pro330 is part of an essential structural and functional element: the flexible catalytic loop suggested to cover the active site as a lid and properly position the catalytic residues. Our investigations provide evidence that Pro330 concurs in the achievement of an optimal catalytic competence. Through a combination of bioinformatic approaches, dynamic light scattering measurements, limited proteolysis experiments, spectroscopic and in solution analyses, we demonstrate that the substitution of Pro330 with Leu, although not determining gross conformational changes, results in an enzymatic species that is highly affected in catalysis with a decarboxylase catalytic efficiency decreased by 674- and 194-fold for the two aromatic substrates. This defect does not lead to active site structural disassembling, nor to the inability to bind the pyridoxal 5’-phosphate (PLP) cofactor. The molecular basis for the pathogenic effect of this variant is rather due to a mispositioning of the catalytically competent external aldimine intermediate, as corroborated by spectroscopic analyses and pH dependence of the kinetic parameters. Altogether, we determined the structural basis for the severity of the manifestation of AADC deficiency in this patient and discussed the rationale for a precision therapy.

**Supplementary Information:**

The online version contains supplementary material available at 10.1007/s00018-022-04343-w.

## Introduction

A common hallmark among pyridoxal 5’-phosphate (PLP) α-decarboxylases of Group II [1] is the presence of a catalytic loop (CL) essential for enzymatic activity [2]. These enzymes play important roles in all tissues and many of them are responsible for the synthesis of important monoamine neurotransmitters. Structurally, they all belong to the aspartate aminotransferase family (or Fold-Type I) [3, 4] of PLP-dependent enzymes and are functional asymmetric homodimers with the CL of each subunit exposed to the solvent and able to insert into the opposite active site in the so-called active closed enzymatic form (Supplemental Results). One of the best characterized among them is mammalian aromatic amino acid decarboxylase (AADC) responsible for the synthesis of dopamine and serotonin from l-3,4-dihydroxyphenylalanine (l-Dopa) and l-5-hydroxytryptophan (l-5HTP), respectively [5]. The spatial structure of pig holoAADC has been solved in the absence and in the presence of the inhibitor carbiDopa (cDopa) [6] that, although irreversibly bound to PLP through its hydrazine group, mimics the substrate binding mode, especially in the positioning of the catechol moiety. The structure of the human apoAADC has also been solved [7] in an unusual open assembly that could account for the mechanism of PLP binding and apoprotein preferential degradation [7]. The global holoAADC architecture is organized in two interlocked monomers, each composed of an N-terminal domain (NTD, residues 1–85), a large domain containing the PLP cofactor (residues 86–360) and a C-terminal domain (CTD, residues 361–480). The CL (residues 328–339) is part of the loop3 (residues 323–357) belonging to the large domain and is assumed to protrude inside the opposite active site during catalysis in the dimeric functional unit [2]. This flexible stretch is visible only in few spatial structures of α-decarboxylases solved so far [2, 8], but not in mammalian AADC. The highly solvent exposed nature of the CL in α-decarboxylases is corroborated by the limited proteolysis susceptibility displayed by AADC [9], human glutamate decarboxylase (GAD) isoforms [10] and human histidine decarboxylase (HDC) [11]. Importantly, the CL contains the catalytic tyrosine residue [12] implicated in the enzymatic mechanism of decarboxylation [8, 11–13] and proposed to be properly oriented toward the Cα of the substrate (as it was later demonstrated for HDC [11] and GAD67 [13] and seen in human cysteine sulfinic acid decarboxylase (CSAD) [2], as well as in plant AADCs [8]). Thus, the flexibility of the CL appears crucial for the orientation of key residues for activity. Among the evolutionarily related α-decarboxylases with solved CL, human HDC shares the highest sequence identity (52.1%) with AADC and has been employed to build a model of the whole pig AADC [14]. Here, it is evident how residues belonging to loop3 such as Asp345 or Arg347 concur in a network of interactions with the CL, to properly position it inside the facing active site [14].

Mutations (substitutions, deletions, insertions, splicing) in the dopa decarboxylase (also called aromatic amino acid decarboxylase) (*DDC*) gene coding for AADC (GRCh38.p13; chr7:50,447,733–50,576,163) lead to AADC deficiency (OMIM#608643), a monogenic neurometabolic disease, determining a complex severe phenotype [15–17] which is often fatal in the first decade. Up to now, over 90 mutations are known and more than 130 patients identified in both homozygosis and heterozygosis [17]. In the last few years, we have characterized more than 30 variants carrying amino acid substitutions [14, 18–23]. Mutations are spread over the entire gene sequence, but none has been identified on the CL until now. Notably, some pathogenic amino acid substitutions map on loop3 [2, 7] and have been already characterized: R347Q [18], R347G [14] and L353P [20]. These variants share an almost invariant structural fold as the wild-type (WT) enzyme, but a remarkable loss of catalytic efficiency attributed to the proximity of the replacements to the active site possibly altering CL engagement into catalysis.

Here, we report the case of a homozygous patient carrying for the first time a substitution, the novel P330L, mapping on the CL of AADC and characterize the structural and functional properties of the P330L AADC protein. Altogether, we propose that the molecular basis for the lack of catalytic activity of the P330L variant consists in the impairment of CL closing to reach a catalytically competent conformation. More precisely, the P330L protein pertains all the structural requirements of the WT counterpart in terms of secondary and tertiary structure as well as in coenzyme binding affinity, but reveals defects in productively orienting the external aldimine catalytic intermediate. This translates into a species unable to produce neurotransmitters at sufficient levels. Patients carrying mutations affecting this element of the protein could be responsive to dopamine agonists and MAO inhibitors with less pronounced benefits from vitamin B6 and L-Dopa treatments and could be possibly considered as candidates for gene therapy having an almost inactive AADC enzyme.

## Materials and methods

### Materials

PLP, l-Dopa, dopamine, cDopa, dopa methylester (DME), L-5-hydroxytryptophan (L-5HTP), serotonin, hydroxylamine hydrochloride, isopropyl-β-d thiogalactopyranoside (IPTG), phenylmethylsulfonyl fluoride (PMSF), trichoroacetic acid (TCA), and protease inhibitor cocktail (P8849) were purchased from Sigma.

### Whole exome sequencing (WES) analysis

Proband’s genomic DNA (isolated from peripheral blood leukocytes) was captured using a SureSelect Human All Exon V7 (Agilent Technologies) exome enrichment kit and paired-end 150-nt sequencing was carried out on an Illumina NextSeq 550 platform (Illumina). Data analysis was conducted using a pipeline developed in the Institute of Mother and Child in Warsaw, Poland. Briefly, the BWA MEM (Burrow–Wheeler Aligner) was used to align reads to the reference genome GRCh38, single nucleotide variants and small insertion/deletions were detected using Genome Analysis Toolkit 4, Haplotype-Caller was used for variant calling and Ensembl Variant Effect Predictor for annotation. Variant status was further evaluated using the population (GnomAD v.3.0) and disease databases (ClinVar and Human Gene Mutation Database) and prediction tools available at VarSome website (https://varsome.com). Variant classification was performed in accordance with the American College of Medical Genetics (ACMG) recommendations [24]. Patient parents signed the informed consent for genetic analysis.

### Sanger sequencing

Sanger sequencing was used as a confirmatory test in proband and in his parents for confirmation of biparental origin of the variant. The primers were self-designed using PRIMER3 software. Primer sequences were available on request. Fluorograms were analyzed using Mutation Surveyor v5.0.1 (Softgenetics).

### Biochemical analysis in the cerebrospinal fluid (CSF), urine and plasma

Collection and handling of CSF samples were performed strictly following a standardized procedure on the basis of former established methods [25]. Reversed-phase HPLC and electrochemical detection were used for measurement of biogenic amine metabolites; organic acid profile in urine was investigated by gas chromatography with mass spectrometric separation after trimethylsilylation for the semi-quantitative determination of vanillactic acid (VLA) and acetyl vanilalanine (AVA) concentrations. Reference ranges for CSF biogenic amine metabolites were obtained using CSF samples taken from patients with neurological disorders without extrapyramidal seizures. The age-dependent metabolite concentration and an appropriately selected control group were used for comparison with the values obtained from our patient.

AADC enzyme activity in serum was measured as previously described [25, 26].

### Site-directed mutagenesis, expression and purification of P330L AADC

WT AADC and P330L variant were obtained as previously described [20]. Mutagenesis reaction was performed using the Quick-Change II kit (Agilent technologies) using the oligonucleotide CCTTTAGACTGGACCTCACTTACCTGAAGC and its complement. All mutations were confirmed by DNA sequence analysis of the whole ORF.

WT AADC and P330L variant were expressed and purified as described in [20]. The enzyme concentration was determined using an ε_M_ of 1.42·10^5^ M^−1^ cm^−1^ at 280 nm. PLP content was determined by releasing the coenzyme in 0.1 M NaOH using ε_M_ of 6600 M^−1^ cm^−1^ at 388 nm [27].

### Apoenzyme preparation and coenzyme binding affinity measurements

Apoenzyme was obtained by incubating 5 μΜ holoenzyme with 15 mM hydroxylamine in 0.5 M potassium phosphate buffer, pH 6.8, at room temperature overnight and loaded on a Desalting 26/10 column (GE Healthcare) preequilibrated with 0.5 M potassium phosphate buffer, pH 6.8, and eluted at 1 mL/min. The eluted enzyme was then concentrated on an Amicon Ultra 15 concentrators (Millipore) and washed with 100 mM potassium phosphate buffer, pH 7.4. The equilibrium apparent dissociation constant for PLP, K_D(PLP)_, was determined as described in [20].

The data were fitted to the following Eq. (1):

1 $$Y = Y_{{{\text{MAX}}}} \frac{{\left[ E \right]t + \left[ {PLP} \right]t + KD\left( {PLP} \right) - \sqrt {\left( {\left[ E \right]t + \left[ {PLP} \right]t + KD\left( {PLP} \right)} \right)2 - 4\left[ E \right]t\left[ {PLP} \right]t} }}{2\left[ E \right]t},$$

where [E]t and [PLP]t are the total concentrations of the enzyme and PLP, respectively, Y refers to the intrinsic quenching changes at a PLP concentration, and *Y*_max_ refers to the fluorescence changes when all enzyme molecules are complexed with coenzyme. Curves fitting was performed using Prism, 8.4.0 (GraphPad), with automatic confidence interval set at 95% and the values obtained are the mean (± standard error of the mean (SEM)) of three independent experiments.

### Kinetic parameters, dopamine detection and coenzyme content determination at different pH values

To determine the kinetic parameters of the decarboxylase reaction, AADC and P330L variant were incubated, respectively, at 5 nM and 3 μM final concentration in the presence of 1 μM and 100 μM exogenous PLP and appropriate different l-Dopa and l-5HTP concentrations. The final reaction volume was 225 μL in 100 mM potassium phosphate buffer, pH 7.4. Reaction times were set to detect a linear product formation. The mixtures were then quenched with 25 μL of a 100% TCA solution. Proteins were precipitated in ice and removed by centrifugation. The supernatants were analyzed by HPLC as described [28, 29] using a Gemini C18 column (150 Å, 4.6 mm, Phenomenex, CA, USA) on a Jasco PU-2080 Plus HPLC system equipped with a UV-1570 detector set at 295 nm. Samples were eluted in 100 mM potassium phosphate, pH 2.35, at a flow rate of 1 mL/min. Standard curves of dopamine peak area were prepared with commercially available dopamine. To determine the kinetic parameters as a function of pH, the buffer was 50 mM bis–tris-propane to avoid ionic strength effects.

2 $$\log y = \log \frac{C}{{1 + H/K_{{\text{a}}} }},$$

where *y* represents either *k*_cat_ or *k*_cat_/*K*_m_, *C* is the pH independent value and *K*_a_ the ionization constant.

To determine P330L coenzyme content in the presence of different substrates (2 mM l-Dopa and 2 mM l-5HTP) or P330L and WT coenzyme content with 2 mM DME, the enzymatic species were incubated with the appropriate substrate/ligand at 10 μM protein concentration in 100 mM potassium phosphate buffer, pH 7.4, at 25 °C. The same analysis was performed for P330L in bis–tris-propane 50 mM buffer at pH 6.5, 7.5 and 8.5. HPLC detection was carried out as above. Standard curves of peak area as a function of coenzyme or cyclic adducts concentration were prepared with commercially available PLP and Pictet–Spengler adduct was obtained by incubating PLP with l-Dopa, l-5HTP and DME, as already described [20]. Experiments were carried out in triplicate and for each measured kinetic parameter the mean ± SEM was reported. Data were fitted to the appropriate Eq. (2) using Prism, 8.4.0, (GraphPad) with automatic confidence interval set at 95%.

### Spectroscopic measurements

All spectral measurements were acquired in 100 mM potassium phosphate, pH 7.4, at 25 °C except for far-UV CD spectra which were recorded in 30 mM potassium phosphate, pH 7.4. CD measurements were recorded with a Jasco J-710 spectropolarimeter at a scan speed of 50 nm/min with a bandwidth of 2 nm at a protein concentration of 1–5 μM. Thermal denaturation was performed by monitoring the CD signal at 222 nm of 4 μM enzyme on a 25–90 °C linear temperature gradient, with a temperature slope of 1 °C/min. 100 μM of exogenous PLP was added to the holoenzymes. Experiments were carried out in triplicate and reported as the mean melting temperature value ± SEM.

Analysis of the external aldimine spectral modifications were carried out on a Jasco V-550 spectrophotometer at a protein concentration of 10 μM in the absence or presence of saturating concentration of substrates l-Dopa and l-5HTP. Fluorescence emission spectra were recorded on a Jasco FP-8500 fluorimeter. Internal aldimine of AADC species as function of pH values was carried out at a protein concentration of 10 μM in 50 mM bis–tris-propane buffer over the pH range 6.0–9.0. Absorbance data were fitted to Eqs. (3) and (4):

3 $$A = \frac{A1 - A2}{{1 + 10^{{{\text{pH}} - {\text{p}}K{\text{spec}}}} }} + A2,$$

4 $$A = \frac{A1 - A2}{{1 + 10^{{{\text{p}}K{\text{spec}} - {\text{pH}}}} }} + A2,$$

where *A*1 and *A*2 are the higher and the lower absorbance limits at a particular wavelength. Fitting to Eqs. (3) and (4) was carried out by using Prism, 8.4.0, (GraphPad) with automatic confidence interval set at 95% and the obtained pKa values represent the mean (± SEM) of three independent experiments.

### Dynamic light scattering (DLS) measurements

DLS measurements were obtained on a Zetasizer Nano ZS instrument (Malvern), using disposable ZEN0112 polystyrene cuvettes. Settings used for particle size measurements were the following: solvent refractive index 1.330, viscosity 0.8872 cP, protein refractive index 1.450, protein absorption 0.001, temperature 25 °C, equilibration 2 min, measurement angle 173 Å backscatter, analysis model multiple narrow modes. Samples were prepared at 3–4 μM protein concentration in 100 mM potassium phosphate, pH 7.4. Native holo forms were preincubated for 30 min with additional 100 μM PLP and 2 mM cDopa-bound species for 30 min before analysis. All samples were filtered using a 0.02 μm Anotop 10 filter (Whatman). Data of particle size are reported as mean ± SEM of three independent replicates. Each single value derives from at least 40 measurements, each one consisting of 12–18 repetitions.

### Limited proteolysis experiments

Limited proteolysis was performed by incubating 0.54 mg/mL AADC (both WT and P330L) with trypsin at the final E/S ratio of 1:100 (w/w) in 100 mM potassium phosphate, pH 7.4 at 25 °C, with the addition of 2 mM DME. Aliquots were withdrawn at different times and compared with AADC sample in the absence of trypsin. At each time point, the reactants were boiled in SDS sample buffer to stop trypsin activity. The digestion products were separated by SDS/PAGE, and gels were stained with Coomassie.

### Bioinformatic analyses

Human AADC sequence (P20711) was aligned with human group II decarboxylase sequences (HDC: P19113, CSAD: Q9Y600, GAD65: Q05329, GAD67: Q99259) retrieved from UniProt database and aligned using Multiple Sequence Alignment software CLUSTALW OMEGA on EMBL-EBI server (http://www.ebi.ac.uk/).

The position and possible interactions of Pro330 as well as the modifications induced by the P330L substitution were analyzed using two approaches. First, we started the homology modeling performed with the Yasara Structure package [http://www.yasara.org/]. Three template structures accessible in PDB (1js3 pig kidney AADC bound to the inhibitor cDopa, 1js6 pig kidney AADC and 4e1o human histidine decarboxylase bound to histidine methylester) were identified automatically. The regions absent in the template structures of pig AADC, including residues 328–339 of the CL, were modeled using knowledge-based loop modeler implemented in the Yasara package. The final hybrid model was built from the best single-template model, in which every sub-optimal region was iteratively replaced with analogous fragments adopted from alternative models. The thermodynamic effect of single-point replacement Pro330Leu was then assessed with the aid of the FoldX program [http://foldxsuite.crg.eu/]. 25 simulation cycles were performed independently for the highest-scored single-template models with open or closed conformation of the CL loop.

Pro to Leu substitution was obtained and minimized using the Protein Preparation Wizard [30] in the program BioLuminate within Schrödinger Suite 2021–4 and applying the OPLS4 force field (BioLuminate, Schrödinger, LLC, New York, NY, 2021). Structural visualization, measurements and figures of the human holoAADC structure were carried out using Pymol 2.2.3 (The PyMOL Molecular Graphics 50 System, Version 2.0 Schrödinger, LLC, New York, NY, 2021). Frustration analysis of the CL was performed by the Frustatometer server [31] for the obtained WT and P330L holoAADC in both the open and closed CL conformations.

## Results and discussion

### Clinical phenotype of a patient carrying the homozygous P330L substitution in the *DDC* gene

A currently 2.5-year-old boy from healthy consanguineous parents, with uneventful pre- and perinatal period and family history was born on time with normal body weight, length and head circumference, and received 10 points on Apgar scale. Since infancy he was unable to suck, and recurrent vomiting with excessive salivation were observed. Since the age of 8 months, he suffered from episodes of anxiety, crying and increased sweating. Since the second year, a generalized weakness had appeared. On pyridoxine 50 mg/d and then 150 mg/d, the patient was quite stable. But at the age of 2 years, he suffered from SARS-CoV2 infection, and oculogyric crises appeared. Folinate was introduced with the initial clinical improvement—he became more active and stronger, with better sleep. Actually, he suffers from marked hypotonia and psychomotor development retardation, but mainly in gross motor skills. Further reduction of the patient’s overall activity and muscle tone was observed in the end of the day with evident improvement after sleeping. Additional analyses of MRI and EEG showed mild, non-specific abnormalities.

### Diagnostic laboratory tests of CSF, urine and plasma of the patient

The CSF biogenic amine metabolite analysis showed reduced concentrations of homovanillic acid (HVA) 133 nmol/L (236–645 nmol/L) and 5-OH-indolacetic acid (5-HIAA) 10 nmol/L (97–367 nmol/L), and an increased concentration of 3-ortho methyl dopa (3-OMD) of 1029 nmol/L (0–50 nmol/L) and L-5HTP 133 nmol/L (0–25 nmol/L). The CSF 5-methyltetrahydrofolate (5-HTF) concentration was normal at 200 nmol/L (78–216 nmol/L) before folinate treatment. Curiously, HVA is relatively high. Interestingly, even if decreased, the HVA level is relatively high with respect to the other measured markers. The urine metabolites of dopamine precursors VLA and AVA were strongly increased in urine organic acid profile; this metabolite pattern is fully characteristic of an AADC deficiency.

The AADC activity in the plasma using l-Dopa as a substrate was 1.0 mU/L (reference range is 16–99 mU/L) and using L-5HTP it was 0.06 mU/L (reference range is 1.0–7.1 mU/L). Thus, AADC enzymatic activity was severely impaired for both substrates.

### Genetic results

The proband’s WES analysis revealed the presence of *DDC* gene variant NM_001082971.2:c.989C > T in exon 10 (p.Pro330Leu) in 74 of 77 reads, indicating its homozygosity, which was further confirmed by Sanger sequencing (Fig. S1A). Biparental origin of the variant was confirmed by Sanger sequencing of the parental DNA (Fig. S1B). The variant was not reported previously according to GnomAD, ClinVar and HGMD databases. In the ACMG classification, it was initially assigned as likely pathogenic, which later, after the completion of in vitro studies, was changed into pathogenic.

### Sequence comparison, modeling and frustration index of the CL for AADC reveal that Pro330 concurs to the functional flexibility of the CL

The sequence alignment of the CL region of human Group II α-decarboxylases (Fig. S2) shows that Pro330 is conserved in AADC and HDC or substituted by an alanine residue in CSAD and in the two GAD isoforms, highlighting that a small aliphatic residue is requested in that position. A model of the loop of AADC was already built from the coordinates of the solved pig enzyme modeling the solved loop region of the homologous human HDC [14]. The active site organization with the relevant residues playing a role in the PLP binding cleft and substrate aromatic side chain positioning have been already identified [2]. The CL is presumed to be highly flexible, and a Pro330 residue could serve as a hinge and thus dictate the freedom degrees of the CL to position the catalytic Tyr332 [12].

**Fig. 1:**
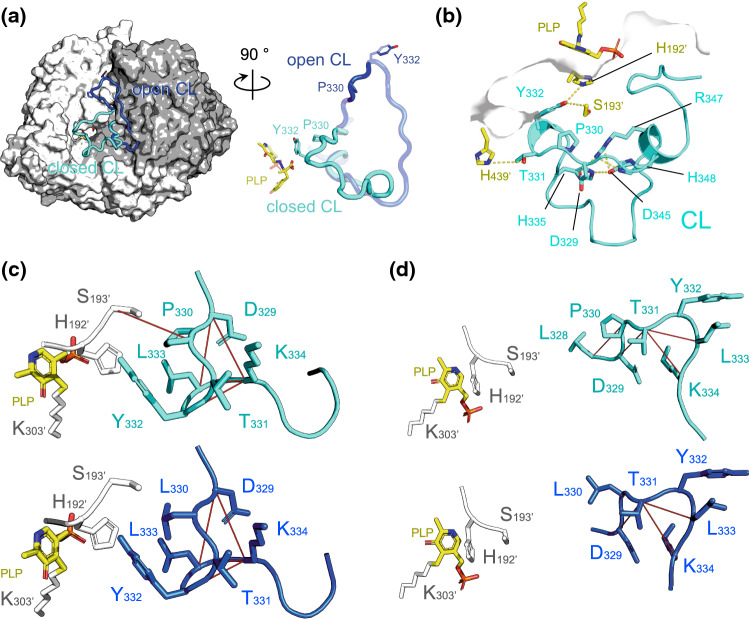
Modeled catalytic loop (CL) of AADC. **A** Dimeric AADC is shown as surface with the two monomers colored white and gray. PLP, cDopa, Pro330 and Tyr332 are shown as sticks. The modeled closed CL (cartoon tube, cyan) of one monomer is inserted into the active site of the other monomer with Pro330 and Tyr332, both in close proximity to PLP of the facing monomer, while the modeled open CL (cartoon tube, blue) is completely solvent exposed. **B** The closed CL is favored by several interactions among residues of CL (in cyan), of loop3 (in cyan) and of the neighboring monomer (in yellow, the prime, ‘, denotes residues of the opposite subunit). Frustration of the CL in both the closed **C** and open **D** conformation calculated for the WT (monomer A showing the CL in cyan, monomer B in white, PLP in yellow) and P330L (monomer A showing the CL in blue, monomer B in white, PLP in yellow) AADC. Conformational and mutational highly frustrated pairs are represented as red links on the same figure for clarity. The analyses were performed using the Frustratometer server [31, 33] that does not include coenzymes and for this reason PLP has been shown for clarity. As above, residues belonging to the opposite monomer of the CL are indicated with the prime

Starting from the pig AADC structure and HDC loop region, we obtained a few possible CL conformations where Pro330 approaches the active site of the opposite monomer, as already suggested [6, 12, 14]. Although this loop seems to adopt the closed conformation in the highest-scored model, also open conformations are observed in some others. In general, all models based on native holo pig AADC (PDB 1js6) are in the open conformation and those based on HDC in complex with the ligand histidine methyl ester (PDB 4e1o) are in closed conformation, while those based on cDopa-bound holo pig AADC (PDB 1js3) are either in open or in closed form. These two alternative models, the quality of which was assessed as “good”, are shown in Fig. 1A. In particular, Pro330 is placed inside the active site of the opposite subunit when the CL is in closed conformation, while it is solvent exposed when the CL is open. The thermodynamic effect of the replacement of Pro330 with Leu was assessed for the two highest-scored models, one with both loops in the open conformation and the other with both in the closed form. In both CL conformational states, the P330L replacement was found unfavorable. However, the closed state is distorted much more (2.6 ± 0.2 kcal/mol) than the open one (0.6 ± 0.1 kcal/mol). Thus, upon P330L replacement, the open state of the protein is less disturbed. The difference of about 2.0 ± 0.2 kcal/mol corresponds roughly to a 30-fold shift of the conformational equilibrium toward the CL open state.

Following this view, the P330L replacement is expected to affect the positioning of the loop.

A deep inspection reveals that Pro330 represents a conformational constraint that allows the CL to bend and properly position Tyr332 bridging it to both His192’ and Ser193’ (the prime denotes residues of the neighboring subunit) (Fig. 1B). The bending is reinforced by a number of H-bonds concurring in holding in place the loop: Arg347 is involved in an H-bonding network with Asp345 and His335. On the opposite side, the CL is maintained into the active site groove by interactions with a structural motif (a beta sheet, residues 436–441) belonging to the CTD distant 4–5 Å from the flexible stretch, mainly by Thr331 reaching His439’. It is reasonable to propose that the substitution of Pro330 with Leu would lead to a loosening of the conformational role played by Pro and although the chemical nature of the residue is not altered, its conformational freedom could be affected, with possible consequences on Tyr332 proper positioning. To gain some insight into the setting/twisting of the unstructured CL into the opposite active site, we computed the frustration index of residue in the Pro330 and Leu330 AADC proteins. The frustration notion, arising from statistical physics and applied to biomolecules [32], is suitable to evaluate the conformational “freedom” of highly flexible regions [33]. The single residue frustration index of Pro330 is below -1 only for one monomer in the closed CL state (Table 1) and in the other cases it is neutrally frustrated. A substituted Leu in position 330 increases the frustration index, thus increasing the energetically favorable interactions for both the subunits in the open and closed CL state. This could mean that the mutated residue has no gross energetic disadvantage in itself. Then, we calculated the mutational and conformational frustration index of the contact pairs involving the residue at position 330 and its related network (Fig. 1C and D). The total number of highly frustrated interactions increases in mutational frustration when Leu is present at the place of Pro330 in the closed CL state. In configurational frustration, the number of highly frustrated interactions between WT and P330L variant is equal when the CL is in the closed state and decreases in the P330L with respect to the WT by one unfavorable interaction in the CL open state. Thus, the Pro-to-Leu substitution leads to a less frustrated open CL.

**Table 1:** Single-residue, mutational and configurational frustration level index of Pro330 (in the WT AADC) and Leu330 (in the P330L AADC variant) in the open and closed conformation of the CL for the two monomers of AADC

	Single residue	Mutational^a^	Configurational^a^
WT			− 1.988 (residues 328’–330’)
CL open		–	− 1.62 (residues 329’–331’)
Monomer A	− 1.036		− 1.643 (residues 331’–333’)
Monomer B	− 0.719		− 2.714 (residues 331’–334’)
CL closed		− 1.114 (residues 193–330’)	− 2.534 (residues 329’–333’)
Monomer A	− 0.928		− 1.199 (residues 329’–334’)
Monomer B	− 0.702		− 1.686 (residues 331’–334’) − 1.118 (residues 332’–334’)
P330L			− 1.662 (residues 329’–331’)
CL open		–	− 1.682 (residues 331’–333’)
Monomer A	1.188		− 2.722 (residues 331’–334’)
Monomer B	0.912		
CL closed		− 1.248 (residues 329’–334’)	− 2.459 (residues 329’–333’)
Monomer A	1.162	− 1.149 (residues 331’–334’)	− 1.181 (residues 329’–334’)
Monomer B	0.912		− 1.668 (residues 331’–334’) − 1.102 (residues 332’–334’)

^a^The prime (’) denotes residues from the neighboring subunit

Overall, the in silico analysis indicates the importance of Pro330 in keeping the CL well fitted to orient the catalytic residue Tyr332 properly.

**Fig. 2:**
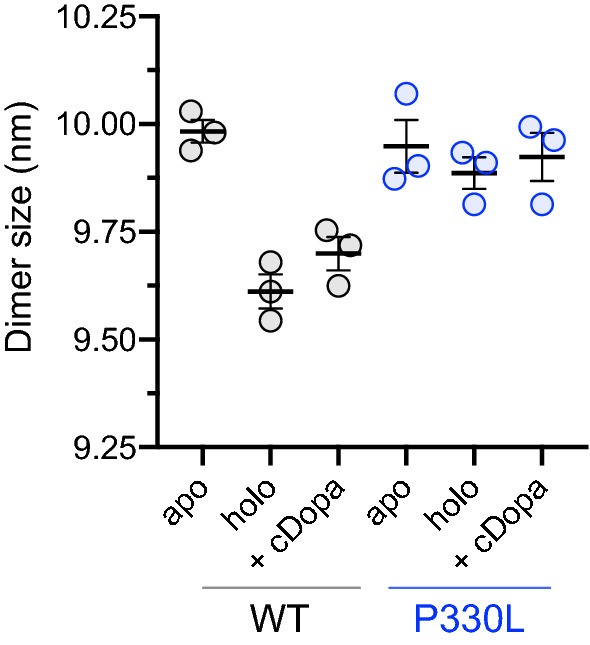
Hydrodynamic diameters calculated with DLS. Particle size relative to apo and holo AADC species as well as the protein bound to the inhibitor cDopa. Results derive from multiple runs of three independent experiments and are reported as mean ± SEM

Interestingly, the dimer hydrodynamic radius of apoWT is greater than those of the holoWT and its cDopa-bound form (Fig. 2 and Table 2). A slight difference is observed comparing holoAADC in the absence or presence of cDopa. It is not unprecedented that a small difference in compactness could give rise to small changes in this parameter, as in HDC whose oxidized more compact form is slightly smaller than the reduced one [34] due to the presence of only one additional disulfide bridge. Meanwhile, apoP330L radius is comparable to the apoWT one and does not evidence differences in apo-, holo-, and cDopa-bound state. This reinforces the fact that P330L variant is insensitive to possible modifications of compactness induced by coenzyme or ligand binding.

**Table 2:** Hydrodynamic radius of apo, holo and cDopa-bound WT and P330L AADC in 100 mM potassium phosphate, pH 7.4

Enzyme	WT			P330L		
Species	apo	holo	+ cDopa	apo	holo	+ cDopa
Mean (nm) ± SEM	9.98 ± 0.02	9.61 ± 0.04	9.70 ± 0.04	9.95 ± 0.06	9.89 ± 0.04	9.92 ± 0.05

^a^Data are reported as mean ± SEM, expressed in (nm) and are obtained from three independent replicates (see “Materials and methods” for details)

### The structural features of P330L AADC are not affected by amino acid substitution but CL flexibility is compromised

**Fig. 3:**
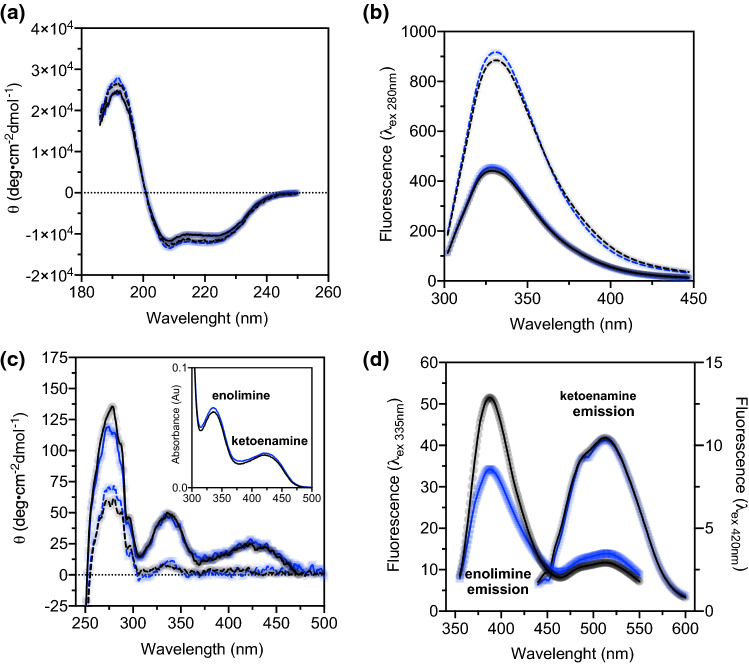
Spectroscopic analysis of WT and P330L AADC species. HoloWT (black solid line), apoWT (black dashed line), holoP330L (blue solid line) and apoP330L (blue dashed line) AADC species were analyzed by means of spectroscopic techniques. **A** Far-UV CD spectra were recorded at 1 μM protein concentration in 30 mM potassium phosphate buffer, pH 7.4, with the addition of 100 μM PLP for the holo forms. **B** Intrinsic emission fluorescence recorded upon excitation at 280 nm in 100 mM potassium phosphate buffer solution at pH 7.4 at 1 μM protein concentration. **C** Near-UV CD spectra of apo and holo AADC species recorded at 5 μM protein concentration in 100 mM potassium phosphate buffer pH 7.4 with the addition of 100 μM PLP for the holo forms. The inset shows the visible absorbance spectra of 10 μM holo protein. **D** Bound coenzyme emission fluorescence spectra of 1 μM WT and P330L AADC recorded in 100 mM potassium-phosphate buffer at pH 7.4 upon excitation at 335 nm (for enolimine emission) and 420 nm (for ketoenamine emission)

The far UV CD spectra of apo and holo P330L show a similar profile and secondary structure content and are not different from the corresponding ones of the WT suggesting that the overall fold is not affected by the amino acid substitution (Fig. 3A). This is also mirrored by similar values of thermostability at 222 nm (Table 3). Moreover, intrinsic fluorescence (Fig. 3B) and near UV (Fig. 3C) spectra of P330L of both holo and apo enzymes are well superimposable to those of the WT, suggesting that the amino acid alteration on the highly mobile CL element has not determined changes in terms of tertiary structure in the protein. Notably, the visible CD (Fig. 3C) and absorbance spectra (inset Fig. 3C) of P330L AADC present the same signals associated with the tautomeric equilibrium of PLP bound to the active site Lys303 in forms of enolimine and ketoenamine tautomers as the WT [35], suggesting that the PLP binding mode is not altered by substitution. However, while the fluorescence emission of the ketoenamine tautomer of P330L is identical to that of the WT (*λ*_max_ = 513 nm), the emission signal of the P330L enolimine fluorophore displays a smaller quantum yield (about 70%) with an identical emission maximum (*λ*_em_ = 387 nm) concomitant with a small increased contribution of the 513 nm emission signal due to energy transfer to the ketoenamine tautomer [36], revealing that the enolimine emission is somewhat quenched and the 3’-OH proton transfer to the aldimine nitrogen is more favored in P330L AADC than in the WT enzyme. This is indicative of a subtle dissimilar microenvironment in the variant such as a change in the PLP microenvironment [37]. The attribution of the 335 nm species to the enolimine tautomer is corroborated by the fact that (i) the apoenzyme does not display fluorescence signal when excited at 335 nm (data not shown) and (ii) the excitation spectra of the species that are emitted at about 520 nm and 390 nm are due to a component whose excitation spectrum is centered at 347 nm for WT and 343 nm for P330L (Fig. S4) that could be more properly related to a sp2-carbon, since a possible sp3 carbon of a carbinoamine or a gem-diamine would have contributed at more blue-shifted wavelengths [38]. In addition, these spectra show slight differences in wavelength maxima of P330L with regard to the WT, still reinforcing the idea of altered PLP orientation.

**Table 3:** Thermostability at 222 nm of holo-, apo- and cDopa-bound WT and P330L AADC in 100 mM potassium phosphate buffer, pH 7.4

AADC	WT (°C)	P330L (°C)
Apo	62.8 ± 0.1	62.4 ± 0.1
Holo	67.7 ± 0.2	68.3 ± 0.4
+ cDopa	68.9 ± 0.4	69.9 ± 0.3

Experiments were carried out as described under “Materials and methods”. Results are reported as the mean ± SEM of three independent experiments

However, this has no consequence on the PLP equilibrium dissociation constant that results in 123 ± 19 nM, a value almost identical to that of the WT (101 ± 10 nM) measured under the same experimental conditions.

**Fig. 4:**
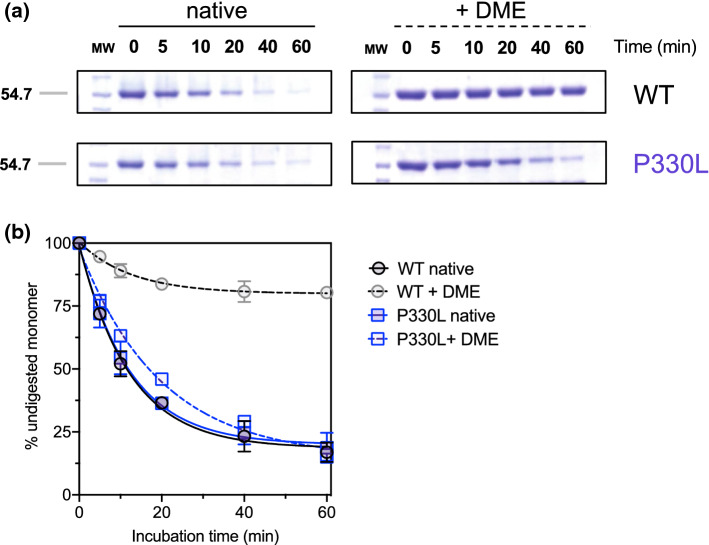
Limited proteolysis of native- and inhibitor-bound AADC species. Limited proteolysis was carried out with a trypsin:AADC ratio of 1:100 (w/w) in the presence of 100 μM PLP alone or 2 mM DME in 100 mM potassium phosphate, pH 7.4 at 25 °C. At various time intervals, aliquots were withdrawn and subjected to SDS-PAGE. **A** The lanes refer to the incubation times, MW = molecular weight markers. **B** Time course of trypsin digestion of native AADC (black circles), native P330L (blue squares), both WT (gray circles) and P330L (light blue squares) in the presence of DME. Species are expressed as a percentage of initial undigested monomer. The rate values are reported as mean ± SEM of three independent experiments

Given the proximity of the substituted amino acid to the tryptic site (Lys334-His335 bond) of the WT enzyme [9], we determined the trypsin accessibility to P330L with respect to the WT in the unliganded form or complexed with DME, the esterified form of l-Dopa unable to undergo decarboxylation [14]. While both unliganded WT and P330L show a rate of proteolysis of about 0.082 min^−1^ under the same experimental conditions, DME-P330L proteolysis slightly slows down (0.036 min^−1^). WT-DME, instead, shows a marked protection from digestion (Fig. 4A and B), thus suggesting that a Leu residue in that position does not dramatically alter CL exposure in the absence of a ligand, but eventually exerts its effect on CL flexibility, possibly hindering loop closure upon the active site. In this sense, limited proteolysis data suggest that the task of Pro330 is to concur in governing CL flexibility.

### The kinetic competence of P330L is highly compromised

When P330L AADC variant was incubated with both substrates L-Dopa and L-5HTP (for details see under “Materials and methods”), a drop in the catalytic activity is observed. Table 4 shows the kinetic parameters and evidences how the highly affected activity versus l-Dopa (0.15% in terms of catalytic efficiency) seems to be driven mainly by a decrease in *k*_cat_ (0.5% that of the WT), while *K*_m_ increases by 3.5-fold that of the WT. A similar behavior is seen with L-5HTP with *k*_cat_ resulting in 1.6% that of the WT, while *K*_m_ is increased 3.2-fold leading to a catalytic efficiency of 0.5% that of the WT. Thus, the P330L substitution compromises catalysis rather than overall protein structural features.

**Table 4:** Kinetic parameters of WT and P330L AADC in 100 mM potassium phosphate buffer, pH 7.4 at 25 °C

Substrate	Enzyme	*k*_cat_ (s^−1^)	*K*_m_ (mM)	*k*_cat_/*K*_m_ (s^−1^ mM^−1^)
L-Dopa	WT	5.5 ± 0.1	0.016 ± 0.001	344 ± 22
	P330L	0.0291 ± 0.0004	0.057 ± 0.002	0.51 ± 0.05
L-5HTP	WT	0.56 ± 0.02	0.009 ± 0.001	62 ± 7
	P330L	0.0092 ± 0.0003	0.029 ± 0.003	0.32 ± 0.03

Data are reported as mean ± SEM of three independent experiments

**Fig. 5:**
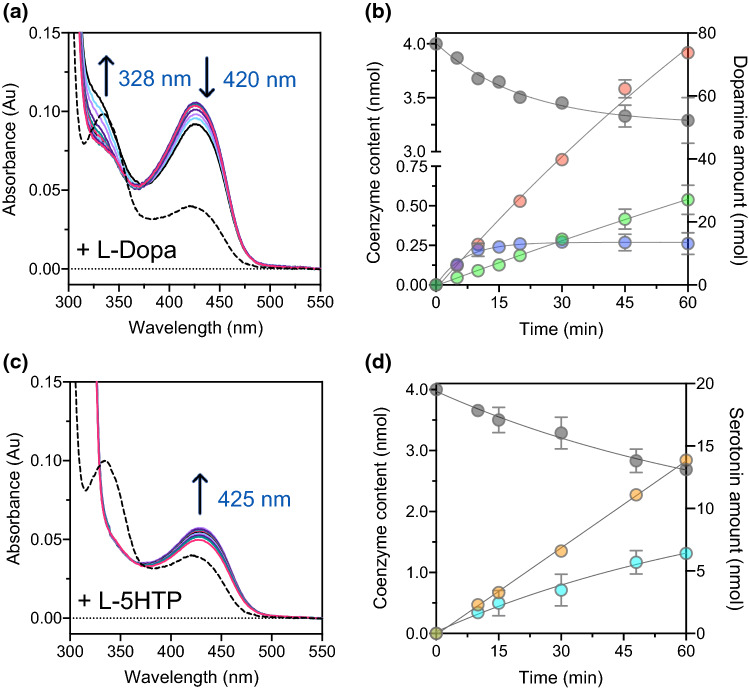
Absorbance spectral changes and coenzyme content modification during the reaction of P330L AADC with L-Dopa and L-5HTP. All reactions were carried out in 100 mM potassium phosphate, pH 7.4 at 25 °C for 1 h. **A** Spectral modification of 10 μM P330L variant in presence of 2 mM L-Dopa. The arrows indicate the increase at 328 nm and the concomitant decrease at 420 nm with time. **B** HPLC analyses of the reaction mixture of 10 μM P330L in presence of 2 mM L-Dopa. PLP (gray circles), PLP-L-Dopa adduct (blue circles), PMP (green circles) and dopamine (orange circles). **C** Spectral modification of 10 μM P330L variant in the presence of 2 mM L-5HTP. The arrow indicates the increase at 425 nm with time. **D** HPLC analyses results of the reaction mixture of 10 μM P330L and 2 mM L-5HTP. PLP (gray circles), PLP-L-5HTP adduct (cyan circles), and serotonin (orange circles). Dashed lines represent the absorbance spectra in the absence of the substrate. The values are reported as mean ± SEM of three independent experiments

To unravel the molecular reason for the loss of catalytic competence, we carried out a spectral analysis of coenzyme modification upon substrate binding. The addition of 2 mM l-Dopa to 10 μM P330L AADC in potassium phosphate buffer, pH 7.4 at 25 °C, determines an immediate increase at 420 nm that slowly decreases with time concomitant with an increase at about 328 nm (Fig. 5A). Interestingly, the WT at the same pH value shows different spectral signals with a 420 nm band converting into a 390 nm absorbing species attributed to a more reactive external aldimine form [39, 40] (see below). HPLC analysis of the reaction mixture reveals that about 20% of the coenzyme decreases (Fig. 5B), being converted into 15% of pyridoxamine 5’-phosphate (PMP) (arising from the ability of AADC to catalyze multiple side reactions in addition to the main decarboxylation one [41–44], as evidenced in other AADC variants [20]) and 5% of the Pictet–Spengler adduct, a cyclic compound formed by spontaneous non-enzymatic nucleophilic addition of the aromatic ring of the aromatic substrate to the nitrogen atom of the external aldimine complex [9]. The formation of this compound depletes the original PLP content and leads to enzyme inactivation. Its recovery is a hallmark of coenzyme exposure to the solvent and has been already detected in several AADC deficiency variants [19, 20], especially when compromised catalysis is due to structural disassembling. This is not the case for P330L that, as other so-called catalytic variants such as L353P [20], forms very low amounts of cyclic compound not responsible for the measured drop of activity.

The addition of 2 mM l-5HTP to 10 μM P330L AADC in potassium phosphate buffer, pH 7.4, at 25 °C leads to a slow increase at 420 nm that continues to increase with time (Fig. 5C). The HPLC analysis shows that PLP slowly decreases, being converted into PLP-L-5HTP cyclic adduct, and serotonin linearly increases (Fig. 5D).

Altogether, the marked decrease in catalytic efficiency of P330L is not driven by a gross protein structural change or by an opening of the active site, since the cyclic adduct formation (signature of PLP exposure to the solvent) is kept at negligible levels.

### The substitution of Pro330 with Leu affects the conformation of the internal aldimine that triggers a dropped kinetic capability of the external aldimine

**Fig. 6:**
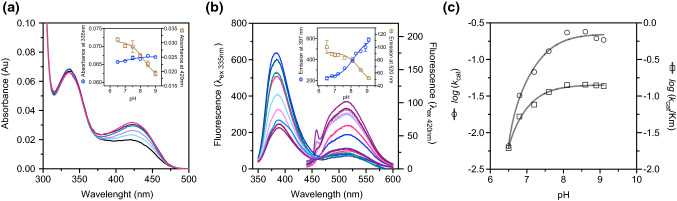
pH dependence of the absorbance and fluorescence bands of P330L internal and WT and P330L external aldimine with DME and of the catalytic parameters for the P330L decarboxylation of L-Dopa. **A** Absorbance spectra of 10 μM P330L in 50 mM bis–tris-propane buffer at different pH values. The inset shows the pH dependence of the enolimine (335 nm, circles)) and ketoenamine (420 nm, squares) tautomers. Solid lines represent the theoretical fitting according to Eq. (3) and (4). **B** Coenzyme emission fluorescence spectra of 1 μM P330L in 50 mM Bis–Tris-propane buffer at different pH values upon excitation at 335 nm (*λ*_em max_ = 387 nm) and 420 nm (*λ*_em max_ = 513 nm). The inset shows the theoretical fit of the experimental data of pH dependence of the enolimine (387 nm, circles) and ketoenamine (513 nm, squares) species. Solid lines represent the theoretical fitting according to Eq. (3) and (4). **C)** log *k*_cat_ (left axis) log *k*_cat_/K_m_ (right axis) for the decarboxylation of L-Dopa by P330L AADC. The curves are obtained by fitting the experimental points to Eq. (2). Data are reported as the mean of three independent experiments and the standard error was less than 10%. Experimental details are reported under “Materials and methods”

Up to now, the only clue indicating an alteration at the active site microenvironment triggered by the Pro-to-Leu substitution is the anomalous behavior of the enolimine tautomer of the internal aldimine and the absence of the external aldimine with L-Dopa absorbing at 390 nm typical of the WT and associated with more efficient catalysis [39, 40]. Firstly, we determined the pH dependency of the internal aldimine absorbance bands to investigate the tautomeric equilibrium. The equilibrium between the ketoenamine and the enolimine tautomers of WT AADC has been already determined and attributed to a residue with p*K*_a_ of about 7.3 controlling it [40]. Interestingly, the equilibrium distribution of tautomers of the internal aldimine of P330L as a function of pH shows that the ketoenamine absorbance band displays a p*K*_a_ of 8.2 ± 0.2, while the enolimine absorbance signal seems to be mostly insensitive of pH (Fig. 6A and inset). If the fluorescence emission of P330L is evaluated upon excitation at 420 nm as a function of pH, the emission of the ketoenamine at 513 nm shows a pH dependence with a measured pKa (8.5 ± 0.2) (Fig. 6B and inset) that overlaps with the value obtained in absorbance. Curiously, if excitation is set at 335 nm, the 387 nm emission increases with pH with a p*K*_a_ of 8.3 ± 0.1 concomitantly with the decrease of the 513 nm emission band (Fig. 6B and inset). Since fluorescence probes the excited states while absorbance the ground states, it should be inferred that P330L substitution leads to a proton shift competition between the 3’-OH of the excited state of the enolimine and the aldimine nitrogen of the ketoenamine excited state under the control of an acid/base group that influences the tautomeric equilibrium. A role for such a catalytic acid–base chemistry could be advanced for Thr246 placed near the 3’-oxygen of PLP [6] and in contact with His192 and Ser193 which, in turn, are strictly connected to the CL. Thus, the different p*K*_a_ value of the residue/s controlling the internal aldimine tautomeric equilibrium in P330L with respect to the WT could be a consequence of CL misplacing and should be mirrored by some alteration in the catalytic external aldimine intermediate when the substrates bind the coenzyme and start the catalytic process.

Interestingly, the P330L variant in the presence of L-Dopa forms a 420 nm absorbing band (not converting into a 390 nm species over the entire pH range as, instead, the WT accumulates [39, 40]) that decreases with time concomitant with an increase at 328 nm (Fig. S3). By loading onto HPLC the reaction mixtures at pH 6.5, 7.5 and 8.5, it can be observed that dopamine is linearly formed during 1 h with concomitant small conversion of the PLP cofactor into PMP and Pictet–Spengler adduct that globally deplete less than 20% of the total coenzyme (Fig. S3). Thus, these coenzymatic species are not responsible for low activity of P330L.

The anomalous catalytic behavior of P330L AADC is also confirmed by the dependence of the catalytic parameters on pH. Both log *k*_cat_ and log *k*_cat_/*K*_m_ plot increase as a function of pH with a pKa of 6.9 ± 0.1 and 7.0 ± 0.2, respectively (Fig. 6C). Since this p*K*_a_ value is present in both plots, it can be assigned to a residue (probably the same) essential for catalysis. Interestingly, this pH dependence is rather different from that exhibited by the WT enzyme where the acidic p*K*_a_ value on the log *k*_cat_ and log *k*_cat_/*K*_m_ plots (p*K*_a_ around 6.3) was attributed to the 4-*N*’-deprotonation of the external aldimine leading to the 390 nm species [40]. Since in P330L the 390 nm external aldimine does not form, it is reasonable to attribute the p*K*_a_ of 7 to a residue of the enzyme implicated in catalysis. The easiest attribution is His192 implicated in deprotonating the catalytic Tyr332, as suggested [35, 44]. The same could be proposed also to the p*K*_a_ of about 6.3 of the WT AADC. The increase of 1 pH unit mirrors that of the internal aldimine and should be a direct consequence of alteration of polarity of the microenvironment/catalytic intermediate positioning in the P330L variant.

The absence of the 390 nm absorbing species that was attributed to an enolimine species [40, 44] more reactive than the 420 nm ketoenamine tautomer (that builds up, but is rapidly converted into the 390 nm species at physiological pH [39] and is the only form present at acidic pH for the WT [40]) led us to consider if the binding mode of external aldimine is somehow altered in the P330L variant.

**Fig. 7:**
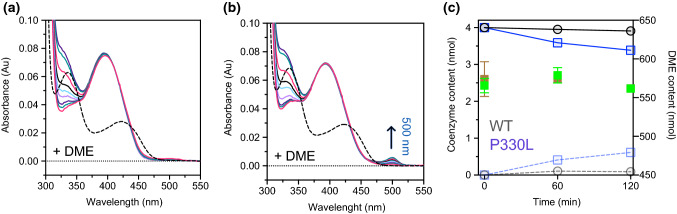
Absorbance spectra and HPLC analyses of the reaction of either WT or P330L AADC with DME. **A** Absorbance spectra of 10 μM WT AADC before (dashed line) and after the addition of 2 mM DME (colored lines) in 100 mM potassium phosphate, pH 7.4 at 25 °C for 2 h; **B** absorbance spectra of 10 μM P330L AADC before (dashed line) and after the addition of 2 mM DME (colored lines) in 100 mM potassium phosphate, pH 7.4 at 25 °C for 2 h; **C** HPLC analyses of coenzyme content following addition of 2 mM DME to 10 μM WT and P330L. Black and light black circles refer to PLP and PLP–DME adduct for the reaction of WT with DME; blue and light blue squares refer to PLP and PLP–DME adduct for the reaction of P330L with DME; brown and green squares represent the total amount of DME in the reaction with WT and P330L, respectively. Lines are drawn only to guide the eye

The addition to WT AADC of 2 mM DME determines the appearance of two bands: one absorbing at 398 nm, indicative of the external aldimine intermediate, and the other at 328 nm [45]. While the 398 nm species remains unchanged with time, the 328 nm form increases (Fig. 7A). The spectral modifications could be attributed both to oxidation of the aromatic substrate and to a small amount of the Pictet–Spengler adduct produced, as the HPLC determination reveals (Fig. 7C). When 2 mM DME is added to 10 μM P330L along with a band absorbing at 393 nm and another at 325 nm, another band at 500 nm appears attributable to a quinonoid species as for other variants belonging to the same loop 3 region [14] (Fig. 7B). The HPLC analysis of the reaction mixture evidences a slight propensity for P330L to produce the irreversible cyclic adduct, which is almost absent in the WT (Fig. 7C). This behavior is reminiscent of that of the nicked AADC [9], a species cleaved by trypsin (as reported above) between residues Lys334–His335 on the CL, unable to perform decarboxylation but able to bind and perform other reactions on some aromatic amino acids and or amines or derivatives [44]. In particular, nicked AADC is able to bind DME leading to the formation of a quinonoid species at 500 nm [45]. This was interpreted as a mispositioning of the external aldimine. Here, we can add that each time the CL is somehow displaced by an alteration of the H-bonding network, this leads to catalytic inability enhancing the inherent propensity of PLP to react non-enzymatically with its aromatic substrates/analogs.

## Conclusions

The molecular defect of the novel P330L variant causing AADC deficiency and severe symptoms to the patient can be ascribed to the alteration of the conformational equilibrium between the open and the closed form of the CL, essential for enzymatic function. In the closed catalytically competent conformation of the WT enzyme, Pro330 is conveniently positioned by a broad network of interactions (weak electrostatic and H-bonds) promoted by the interaction of some residues such as Asp329, Asp345, Arg347, and His348 of both extremities of loop3 (residues 323–357) to residues 330–339 of the CL and to some elements of the LD (His192 and Ser193) and of the CTD (His439) as shown in Fig. 1B. In this manner, the cavity of the active site accommodates the CL that is free to enter (closed conformation) and exit (open conformation). In this context, Pro330 seems to act as pivot to allow the CL (and thus Tyr332) to be properly inserted into the active site once the external aldimine is formed, as shown by molecular modeling analyses and protection by proteolysis. The substitution with Leu on one hand destroys the H-bonding network rendering the CL less able to sit in the closed state, on the other it influences internal and external aldimine placing, thus affecting catalysis rather than global protein structure. The anomalous polarity and conformation of the internal aldimine, as fluorescence and pH data suggest, are reflected into the external aldimine mispositioning revealed by the absence of the 390 nm band at any pH value in the presence of L-Dopa and the accumulation of a quinonoid species in the presence of the analog DME. The latter is also formed in other AADC pathogenic variants of the loop3 region [14]. This behavior has been proposed to be due to alteration of the H-bonding network governing the positioning of the catalytic residue Tyr332. The great decrease in catalytic efficiency for both substrates does not seem to be due to Pictet–Spengler adduct formation (that would have been indicative of a completely exposed active site [9]), rather to an impaired conformation of the external aldimine detrimental for efficient catalysis. In addition, both the PLP microenvironment and the positioning of the catalytic intermediate evidence alterations in the chemistry of some residues, as witnessed by the different p*K*_a_ values of both the internal aldimine and of the catalytic parameters with respect to the WT. This pathogenic amino acidic substitution has been deeply investigated to unravel the molecular basis for AADC deficiency given the fact that it is mapped on an essential structural and functional element. The obtained results allow for precision diagnosis of AADC deficiency, so for a tailored therapy in the reported patient, instead of trying different medications looking for the best clinical response (as it is usually practiced in AADC deficiency). Recently, a promising precision therapy has been proposed for an AADC deficiency patient by correlation of bioinformatics and biochemical and in solution experiments together with iPSCs validation [46]. Given the severe catalytic impairment exhibited by this novel AADC protein variant, the therapeutic suggestion is to sustain the ongoing clinical management with a dopamine (and serotonin) agonist, to enhance the extremely low endogenous activity. If it appears to be clinically ineffective, gene therapy should be considered.

### Supplementary Information

Below is the link to the electronic supplementary material.

Supplementary file1 (PDF 97 KB)

Supplementary file2 (PPTX 1927 KB)

## Data Availability

Data and material are available upon request.

## Abbreviations

PLP: Pyridoxal 5’-phosphate
PMP: Pyridoxamine 5’-phosphate
CL: Catalytic loop
AADC: Aromatic amino acid decarboxylase
l-Dopa: l-3,4-dihydroxyphenylalanine
l-5HTP: l-5-hydroxytryptophan
DME: l-3,4-dihydroxyphenylalanine methyl ester
cDopa: CarbiDopa
GAD: Glutamate decarboxylase
HDC: Histidine decarboxylase
CSAD: Cysteine sulfinic acid decarboxylase
CSF: Cerebrospinal fluid
HVA: Homovanillic acid
5-HIAA: 5-Hydroxyindoleacetic acid
3-OMD: 3-Orthomethyl dopa
5-HTF: 5-Methyltetrahydrofolate
VLA: Vanillactic acid
AVA: Acetyl vanilalanine
