# Supplementary material for: The novel P330L pathogenic variant of aromatic amino acid decarboxylase maps on the catalytic flexible loop underlying its crucial role
Source: Cell Mol Life Sci. 2022 May 20;79(6):305. doi: 10.1007/s00018-022-04343-w (PMC9121088; doi:10.1007/s00018-022-04343-w)
Supplement: Supplementary file 1 — Supplementary file1 (PDF 97 KB) [file 18_2022_4343_MOESM1_ESM.pdf]

Supplementary Results

Human PLP α-decarboxylases share high identity and a common highly flexible catalytic loop (CL)

Sequence comparison of human PLP α-decarboxylases share high identity (**Table S1**) and a common evolutionary origin [1]. Structure comparison with other enzymes of the same structural family, (Fold-Type I [2] or aspartate aminotransferase family [3]), highlighted some common motifs and structural determinants [4]. Among these, the CL is always present and visible only in few structures. Human glutamate decarboxylase isoform GAD67 [5], human cysteine sulfinic acid decarboxylase (CSAD) (only deposited entry in the Protein Data Bank (PDB) as 2JIS; an analysis using this structure has been carried out in [4]) and human histidine decarboxylase (HDC) [6] show the CL of one monomer protruding into the active site of the other monomer. Interestingly, all the enzymes displaying a visible CL have been solved as dimers in the asymmetric unit. Although the crystal structures are snapshots at low potential energy states, it is evident that GAD67 and CSAD, evolutionary more correlated, display a superposition of the CL similar and slightly different from HDC [4]. Pig aromatic amino acid decarboxylase (AADC) (the only AADC mammalian structure solved until now) [7], human GAD65 [5], mouse CSAD [8] and mouse GADL1 [9] miss this flexible element. In the case of AADC a modeled structure has been built on the basis of the human HDC solved CL structure sharing 52.1% sequence identity and 82.6% sequence similarity by comparison of their mature forms [4] (the full-length amino acid sequences share 51% identity [10] and **Table S1** below).

Table S1-Identity of human PLP α-decarboxylases

#	identity (%)
AADC	--
HDC	51.88
GAD67	24.34
GAD65	22.81
CSAD	21.05

References

1. Sandmeier E, Hale TI, Christen P (1994) Multiple evolutionary origin of pyridoxal-5'-phosphate-dependent amino acid decarboxylases. Eur J Biochem 221: 997-1002. DOI 10.1111/j.1432-1033.1994.tb18816.x
2. Grishin NV, Phillips MA, Goldsmith EJ (1995) Modeling of the spatial structure of eukaryotic ornithine decarboxylases. Protein Sci 4: 1291-1304. DOI 10.1002/pro.5560040705
3. Jansonius JN (1998) Structure, evolution and action of vitamin B6-dependent enzymes. Curr Opin Struct Biol 8: 759-769. DOI 10.1016/s0959-440x(98)80096-1

4. Paiardini A, Giardina G, Rossignoli G, Voltattorni CB, Bertoldi M (2017) New Insights Emerging from Recent Investigations on Human Group II Pyridoxal 5'-Phosphate Decarboxylases. Curr Med Chem 24: 226-244. DOI 10.2174/0929867324666161123093339
CMC-EPUB-79890 [pii]
5. Fenalti G, Law RH, Buckle AM, Langendorf C, Tuck K, Rosado CJ, Faux NG, Mahmood K, Hampe CS, Banga JP, et al. (2007) GABA production by glutamic acid decarboxylase is regulated by a dynamic catalytic loop. Nat Struct Mol Biol 14: 280-286. DOI 10.1038/nsmb1228
6. Komori H, Nitta Y, Ueno H, Higuchi Y (2012) Structural study reveals that Ser-354 determines substrate specificity on human histidine decarboxylase. J Biol Chem 287: 29175-29183. DOI 10.1074/jbc.M112.381897
7. Burkhard P, Dominici P, Borri-Voltattorni C, Jansonius JN, Malashkevich VN (2001) Structural insight into Parkinson's disease treatment from drug-inhibited DOPA decarboxylase. Nat Struct Biol 8: 963-967. DOI 10.1038/nsb1101-963
8. Mahootchi E, Raasakka A, Luan W, Muruganandam G, Loris R, Haavik J, Kursula P (2021) Structure and substrate specificity determinants of the taurine biosynthetic enzyme cysteine sulphinic acid decarboxylase. J Struct Biol 213: 107674. DOI 10.1016/j.jsb.2020.107674
9. Raasakka A, Mahootchi E, Winge I, Luan W, Kursula P, Haavik J (2018) Structure of the mouse acidic amino acid decarboxylase GADL1. Acta Crystallogr F Struct Biol Commun 74: 65-73. DOI 10.1107/S2053230X17017848
10. Sköldberg F, Rorsman F, Perheentupa J, Landin-Olsson M, Husebye ES, Gustafsson J, Kämpe O (2004) Analysis of antibody reactivity against cysteine sulfinic acid decarboxylase, a pyridoxal phosphate-dependent enzyme, in endocrine autoimmune disease. J Clin Endocrinol Metab 89: 1636-1640. DOI 10.1210/jc.2003-031161
