# Supplementary material for: The novel P330L pathogenic variant of aromatic amino acid decarboxylase maps on the catalytic flexible loop underlying its crucial role
Source: Cell Mol Life Sci. 2022 May 20;79(6):305. doi: 10.1007/s00018-022-04343-w (PMC9121088; doi:10.1007/s00018-022-04343-w)
Supplement: Supplementary file 2 — Supplementary file2 (PPTX 1927 KB) [file 18_2022_4343_MOESM2_ESM.pptx]

## Slide 1
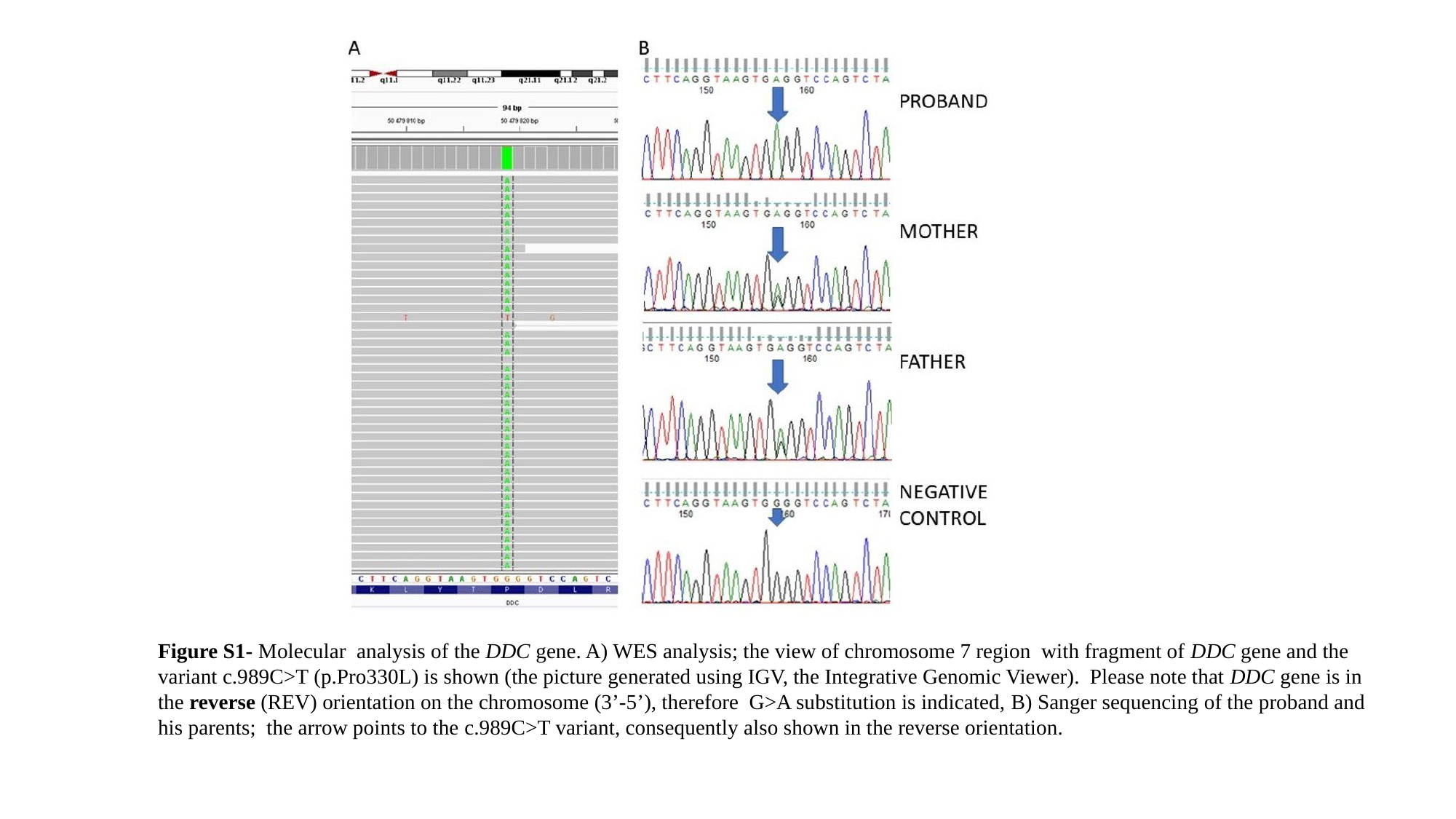

Figure S1- Molecular analysis of the DDC gene. A) WES analysis; the view of chromosome 7 region with fragment of DDC gene and the variant c.989C>T (p.Pro330L) is shown (the picture generated using IGV, the Integrative Genomic Viewer). Please note that DDC gene is in the reverse (REV) orientation on the chromosome (3’-5’), therefore G>A substitution is indicated, B) Sanger sequencing of the proband and his parents; the arrow points to the c.989C>T variant, consequently also shown in the reverse orientation.

## Slide 2
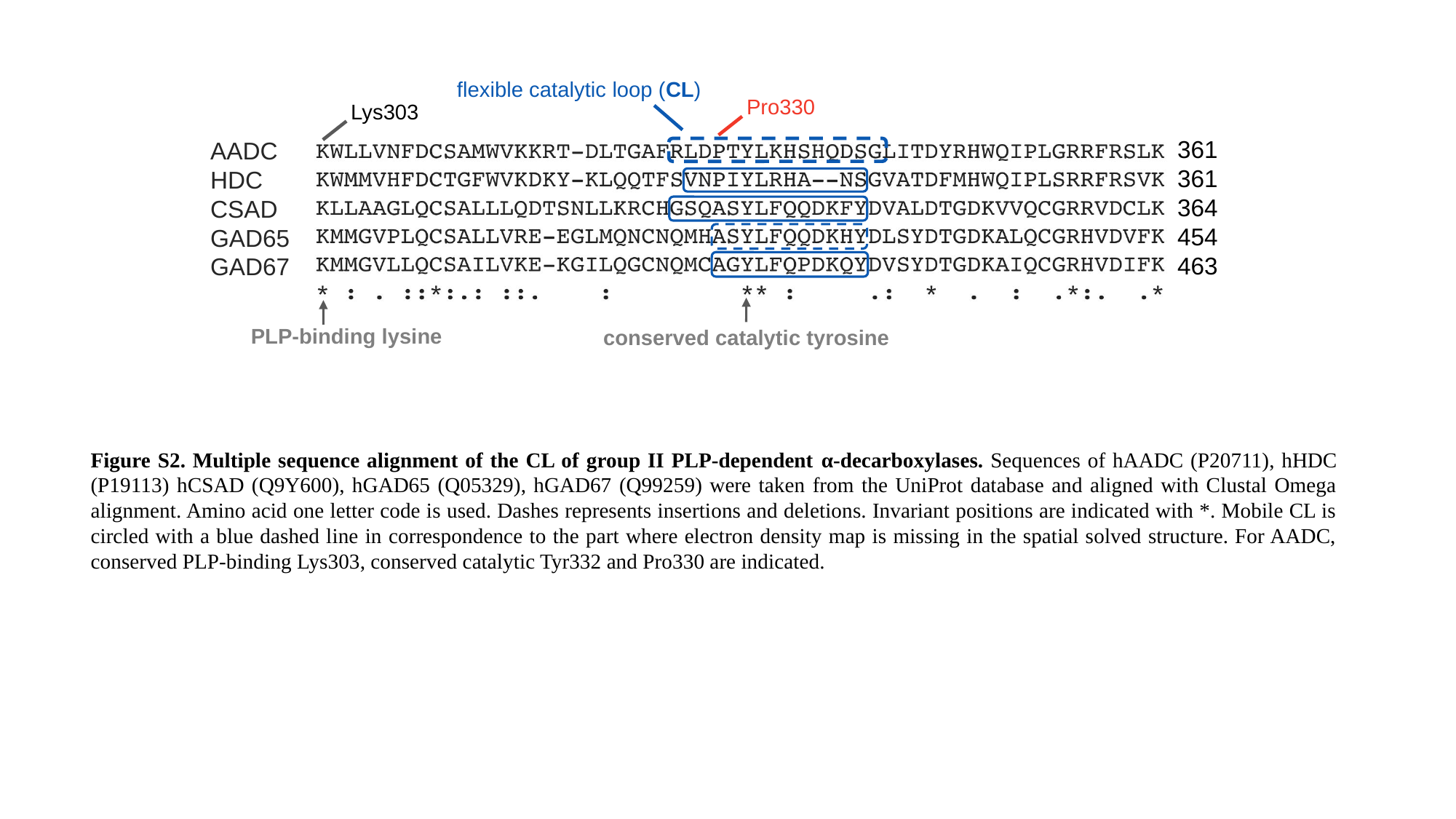

flexible catalytic loop (CL)
Pro330
361
361
364
454
463
AADC
HDC
CSAD
GAD65
GAD67
PLP-binding lysine
conserved catalytic tyrosine
Lys303
Figure S2. Multiple sequence alignment of the CL of group II PLP-dependent α-decarboxylases. Sequences of hAADC (P20711), hHDC (P19113) hCSAD (Q9Y600), hGAD65 (Q05329), hGAD67 (Q99259) were taken from the UniProt database and aligned with Clustal Omega alignment. Amino acid one letter code is used. Dashes represents insertions and deletions. Invariant positions are indicated with *. Mobile CL is circled with a blue dashed line in correspondence to the part where electron density map is missing in the spatial solved structure. For AADC, conserved PLP-binding Lys303, conserved catalytic Tyr332 and Pro330 are indicated.

## Slide 3
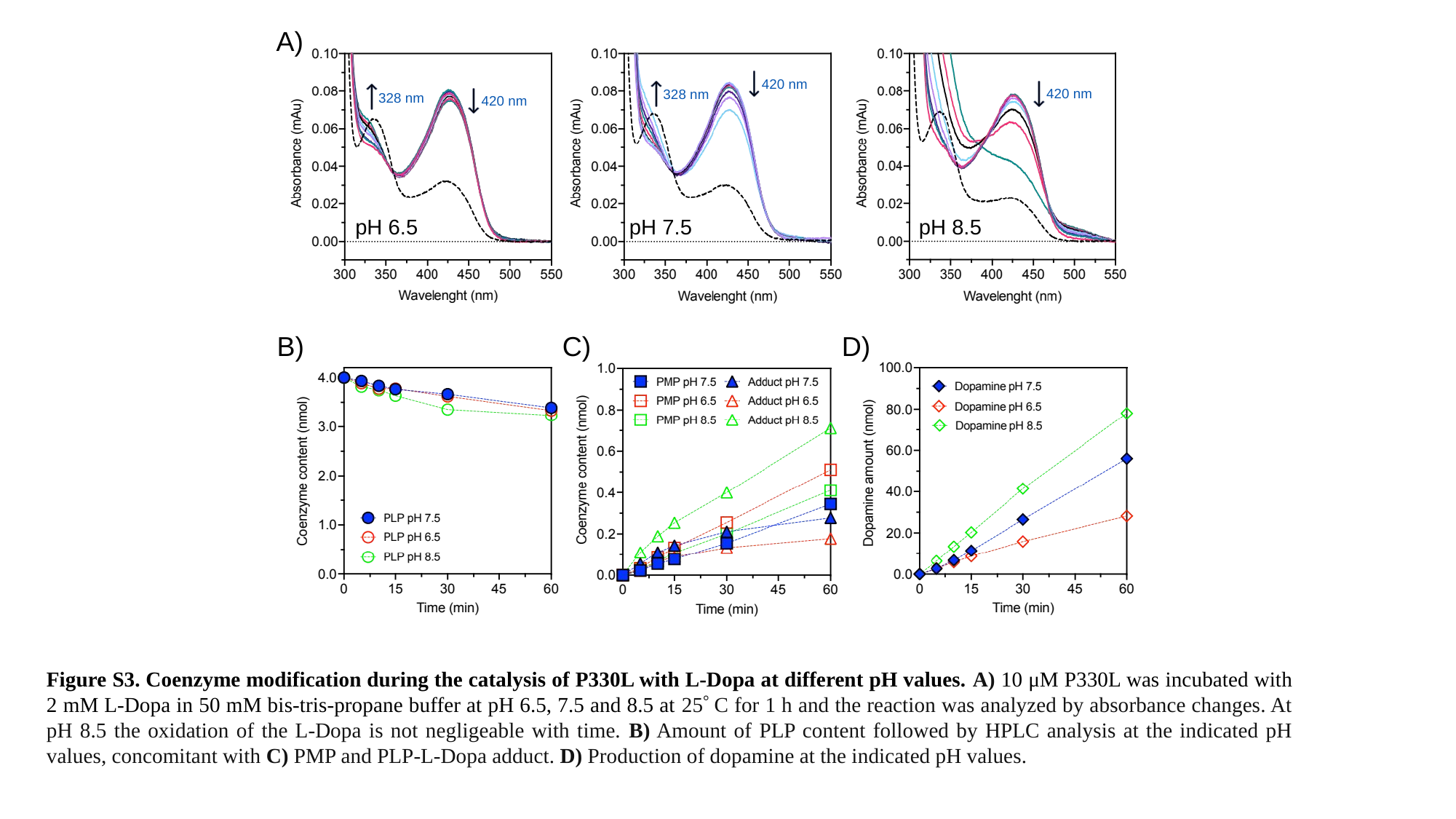

A)
420 nm
420 nm
328 nm
328 nm
420 nm
pH 6.5
pH 7.5
pH 8.5
B)
C)
D)
Figure S3. Coenzyme modification during the catalysis of P330L with L-Dopa at different pH values. A) 10 μM P330L was incubated with 2 mM L-Dopa in 50 mM bis-tris-propane buffer at pH 6.5, 7.5 and 8.5 at 25° C for 1 h and the reaction was analyzed by absorbance changes. At pH 8.5 the oxidation of the L-Dopa is not negligeable with time. B) Amount of PLP content followed by HPLC analysis at the indicated pH values, concomitant with C) PMP and PLP-L-Dopa adduct. D) Production of dopamine at the indicated pH values.

## Slide 4
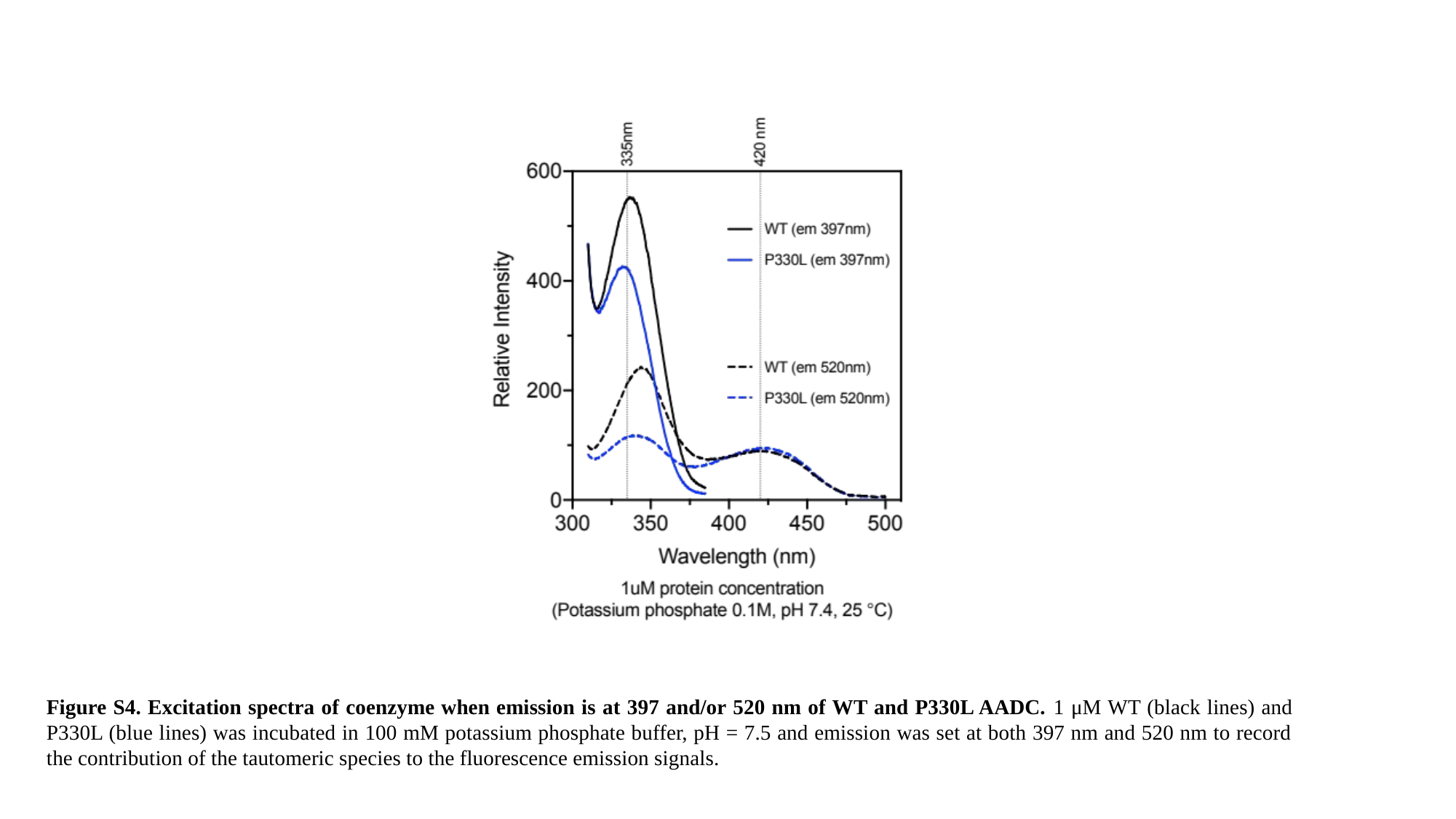

Figure S4. Excitation spectra of coenzyme when emission is at 397 and/or 520 nm of WT and P330L AADC. 1 μM WT (black lines) and P330L (blue lines) was incubated in 100 mM potassium phosphate buffer, pH = 7.5 and emission was set at both 397 nm and 520 nm to record the contribution of the tautomeric species to the fluorescence emission signals.
